# Repeated application of passive mechanical stress produces selective metabolic and extracellular matrix adaptations in human skeletal muscle but does not prevent disuse-induced atrophy

**DOI:** 10.1152/function.109.2025

**Published:** 2026-04-21

**Authors:** Mohadeseh Ahmadi, Charles Seaman, Erik D. Marchant, James Bartling, David Kofoed, Karisa Coombs, Chad R. Hancock, Robert D. Hyldahl

**Affiliations:** Department of Exercise Sciences, Brigham Young University, Provo, Utah, United States

**Keywords:** limb disuse, lipolysis, mechanical stimulation, mechanotransduction, percussive massage

## Abstract

Exposure to mechanical stimuli can modulate skeletal muscle structure and metabolism, yet the extent to which repeated, isolated mechanical stress promotes adaptive remodeling in humans has not been defined. We investigated whether repeated percussive massage (PM)—a widely used but poorly validated therapeutic modality—induces beneficial skeletal muscle adaptations under ambulatory conditions and whether such adaptations confer resilience during limb disuse in humans. In a 6-wk randomized trial, PM did not alter myofiber cross-sectional area, satellite cell abundance, or capillary density, but RNA-Seq pathway analysis revealed enrichment of extracellular matrix (ECM) remodeling networks, which was supported by increases in the expression of basement membrane and focal adhesion components. PM also reduced subcutaneous fat thickness and increased fatty acid-supported mitochondrial respiration while lowering mitochondrial H_2_O_2_ emission. In a separate 10-day immobilization study, PM failed to attenuate unloading-induced reductions in muscle size or strength. However, PM partially preserved fatty acid-supported respiratory capacity relative to a control group, indicating a selective metabolic resilience. Finally, in an acute mechanistic experiment, unilateral PM did not increase subcutaneous adipose tissue lipolysis, as interstitial glycerol concentrations rose similarly in treated and untreated limbs, suggesting that chronic reductions in subcutaneous fat thickness were not driven by lipolytic activation. Collectively, these findings demonstrate that repeated PM promotes targeted skeletal muscle metabolic adaptations, yet is insufficient to induce overt structural remodeling or prevent disuse-induced functional decline.

## Graphical abstract

**Figure:**
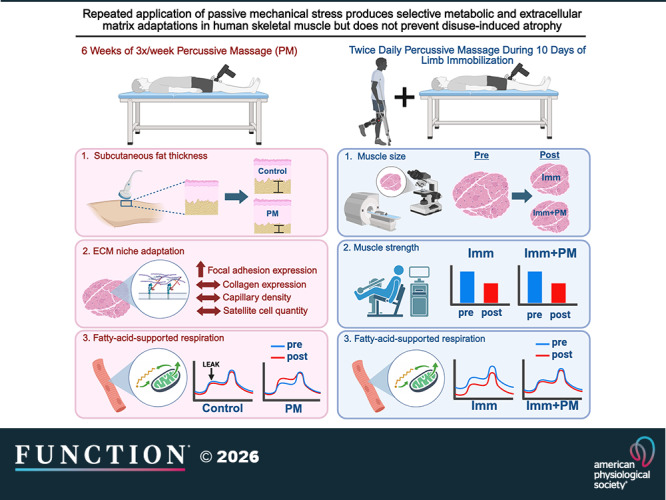

## INTRODUCTION

Skeletal muscle is a highly adaptable tissue capable of extensive structural and functional adaptations in response to a range of physiological and environmental stimuli. These adaptive responses play a critical role in maintaining muscle mass, contractile function, and overall musculoskeletal health through the lifespan. Integrated remodeling of muscle fibers, extracellular matrix, vascular supply, and neuromuscular junctions underpins sustained functional performance and resilience across the lifespan, whereas impaired muscle adaptability is associated with reduced mobility, metabolic dysfunction, and chronic disease (1, 2). Accordingly, skeletal muscle has emerged as a key target for interventions aimed at promoting systemic health, physical performance, and quality of life during aging and disease progression (3).

Exercise training remains the most potent and reproducible intervention to improve skeletal muscle function (4). Exercise training induces adaptation through the collective imposition of a multitude of simultaneous stressors (5). Some of the most prominent and well-studied of which being mechanical (6), hypoxic (7), energetic (8), oxidative (9), and thermal (10). Each of these, when applied to the muscle in isolation is capable of eliciting, through both distinct and overlapping intracellular signaling pathways, improvements in integrative skeletal muscle and organismal health. For example, energetic stress in the form of ATP depletion activates 5′ AMP-activated protein kinase, which in turn activates pathways leading to improved mitochondrial respiratory capacity (11). Likewise, our laboratory has demonstrated that muscle-focused passive heat stress is also capable of improving myofiber respiratory capacity and reducing muscle atrophy in response to limb disuse (12, 13). Studies such as these are important, as resolution of the individual components that drive the health benefits associated with exercise will have therapeutic utility for populations that may be unable to participate in exercise training due to periods of immobilization, disease, or intolerance.

In addition to energetic and thermal stresses, skeletal muscles are also sensitive to isolated mechanical inputs such as stretch, strain, electromagnetic fields, and changes in gravitational forces (6). These mechanical stresses can be transduced through a number of well-described intracellular pathways (e.g., MAPK and FAK) and mechanosensitive ion channels (e.g., Piezo1) (14, 15). In isolated myotube models, for example, mechanical stresses such as stretch, vibration, and the imposition of fluid shear have all been shown to result in a variety of cellular adaptations ranging from improved proteostasis and amino acid uptake to proliferation and differentiation capacity (16–18). However, an underexplored question in the scientific literature is the extent to which repeated bouts (i.e., training) of isolated mechanical stress can drive muscle adaptations that are typically associated with exercise. For example, acute bouts of whole body vibration and stretch have been shown to improve blood flow and decrease blood glucose in whole muscle and isolated myofibers (19, 20). However, whether these acute responses to mechanical stress translate to chronic, beneficial adaptations in humans is unknown.

In this comprehensive study, we sought first to determine some of the primary structural and metabolic adaptations associated with 6 wk of repeated (30 min, 3 times per week) mechanical stress in the form of a recently popular therapeutic modality [percussive massage (PM)]. Next, we asked whether PM was capable of preserving skeletal muscle health and function under a condition that generates pathological degeneration (limb immobilization). As there is relatively little data regarding the effect of repeated PM on muscle adaptation, we first used whole muscle RNA-Seq and gene ontology (GO) analyses in a subset of subjects to guide primary hypothesis development. These initial experiments, combined with the prior preclinical and clinical massage and vibration literature, led us to our general hypotheses—that PM treatment would drive muscle extracellular matrix and microvascular and metabolic adaptation that would contribute to the preservation of functional characteristics under disuse conditions.

## MATERIALS AND METHODS

### Overall Study Description

The analyses presented herein were derived from three distinct interventional trials, each of which were approved by the Brigham Young University Institutional Review Board (IRB2020-309, IRB2022-118, and IRB2023-426) and registered at clinicaltrials.gov (NCT06053229, NCT04733287).

### Study 1

**Figure 1.**
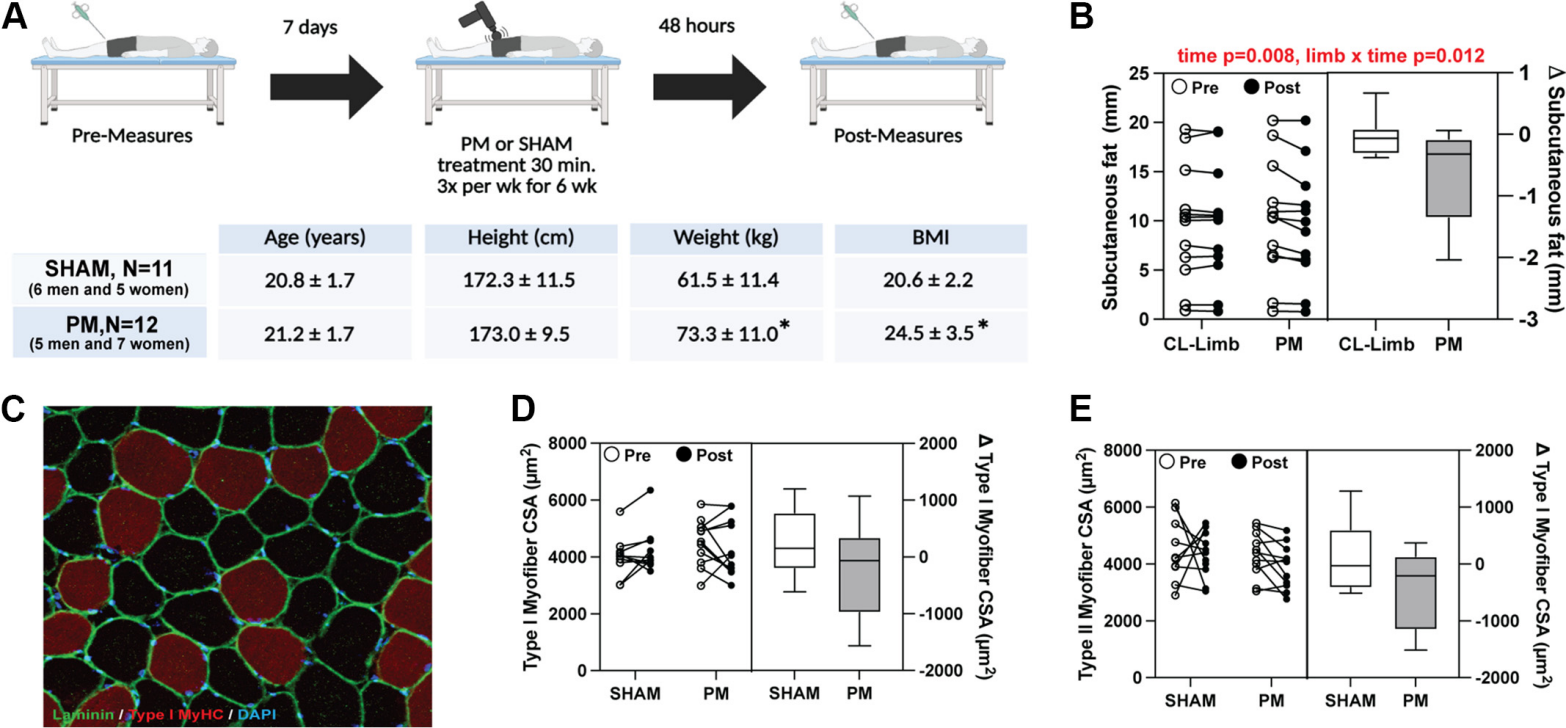
Six-week study design, anthropometric data, and changes in thigh tissue morphology*. A*: schematic of the 6-wk percussive massage (PM) study design and anthropometric data in the SHAM and PM groups. *B*: thigh subcutaneous fat thickness in the treated (PM) and nontreated (CL) limbs (*n* = 12 CL, *n* = 12 PM). *C*: representative image of a skeletal muscle tissue section stained for laminin (green), type I myosin heavy chain (red), and nuclei (blue). Type I (*D*) and type II (*E*) myofiber cross-sectional area (CSA) in the PM and SHAM groups (*n* = 10 SHAM, *n* = 11 PM). Data are presented in a combination panel composed of a paired dot plot with individual subject pre- and postintervention values and a box and whisker plot displaying the distribution of change scores including median, interquartile range, and spread. *P* values are shown in red, where a significant (*P* < 0.05) main effect was found. *Significant difference (*P* < 0.05) between groups.

The first study was a comparative, single-blinded (subjects), SHAM-controlled design to investigate the effects of 6 wk of repeated percussive massage (PM) on skeletal muscle adaptation (Fig. 1*A*). For subjects in the PM group, 12 participants (5 men and 7 women), had a single randomized leg selected for the intervention. The intervention consisted of PM 3 days a week for 30 min per session. The SHAM group consisted of 11 participants (6 men and 5 women), and was part of a separate IRB-approved (IRB2020-023) clinical trial (NCT04733287) designed to determine the efficacy of 6 wk of heat therapy to improve muscle mitochondrial and vascular function. Due to a similar time course, biopsy schedule, study population, exclusion criteria, and outcome measures, this group served as a control group for the current study. Subjects in this group had an identical visit schedule and inclusion/exclusion criteria to those in the PM group. For the SHAM intervention, subjects were asked to lie down, and two short-wave diathermy drums were placed over their quadriceps muscles. However, after the machine was turned on, the heat protocol was not started. Short-wave diathermy produces very little perceptible heat sensation on the skin. To maintain subject blinding, subjects were not told whether they were in the treatment or control group. Subjects in each group were healthy but untrained, defined by not having participated in structured resistance or aerobic training over the past 6 mo. Subject characteristics for study 1 can be found in Fig. 1*A*.

### Study 2

To study the impact of PM on the detrimental effects of limb disuse on skeletal muscle, participants (7 women and 10 men; 18–35 yr) were subjected to 10 days of leg immobilization and unloading and randomly assigned to either the control group (leg immobilization without percussive massage; *n* = 9) or the PM group (leg immobilization with PM; *n* = 8). To qualify for the study, subjects were required to provide positive responses to health screening and engage in regular, structured physical activity (at least 150 min per week). The immobilization protocol was started 14 days after the initial muscle biopsy and pretesting (MRI and dynamometry) to ensure sufficient healing time for the muscle biopsy site. Following the recovery period, the participants started a 10-day phase of leg unloading. The subjects visited the laboratory for their massage/control visits throughout the immobilization period. The massage/control sessions began on the same day as the muscle immobilization protocol. During their first visit to the laboratory, the subjects either received PM on their left thigh (PM group) or lay on the bed (control group) for 10 min. The duration and frequency of massage/control treatments increased over time to prevent hypersensitization to percussive massage. The massage/control visits started with a single 10-min session on the first day and increased to two 20-min sessions on the third day, which continued throughout the limb unloading period. In addition, to help ensure compliance, subjects were required to fill out activity assessment forms beginning 7 days following their initial testing visit until the termination of the study. Twenty-four hours after the final control/massage session, participants returned to the laboratory, still using the knee brace and crutches, to undergo postintervention testing (see Fig. 6*A*). The 24-h interval was selected to allow sufficient time for any acute effects of the final PM bout to dissipate while also permitting participants to resume normal ambulation within a practical timeframe. Subject characteristics for *study 2* can be found in Fig. 6*B*.

### Study 3

The final study was a randomized, controlled laboratory study designed to assess the effects of a single session of PM on subcutaneous fat lipolysis via the measurement of interstitial glycerol concentration (Fig. 8*A*). Participants (5 men and 5 women) had one leg randomly assigned to receive the same 30-min standardized PM treatment, followed by bilateral microdialysis sampling of the subcutaneous adipose layer overlying the vastus lateralis (VL) muscle. Collected dialysate samples were used to assess glycerol and ethanol concentrations. Subject characteristics for *study 3* can be found in Fig. 8*B*.

### Interventions

#### Percussive massage treatments (all studies).

Passive mechanical stimulation for all studies was applied on the participant’s thigh using a 4th generation Theragun pro (Therabody, California). PM was applied on the participant’s thigh, with a reasonable pressure and in a combination of directions, from moving in straight lines to circular movements. The speed of percussion was adjusted between 2,700 and 3,200 rpm according to subject preference and comfort.

#### Limb immobilization (study 2).

Participants were fitted with a therapeutic knee brace (Breg Revolution 3; Brace Direct) and received instructions on how to properly apply and remove it and how to use standard medical arm crutches for ambulation while keeping weight off the designated leg. The left leg of each participant was immobilized with a knee flexion angle of 70°. Participants were instructed not to bear weight on the immobilized leg during the period of leg unloading. Subjects were allowed to remove the immobilization brace at night while they slept and reapply the brace upon getting out of bed.

### Outcome Measurement Methodology

#### Muscle biopsies.

Percutaneous needle biopsies were taken from the middle portion of vastus lateralis of the treated leg using a Bergstrom needle. Biopsies were taken from the treatment leg on the first visit, and 48 h following the final massage session. Using manual suction, ∼75–150 mg of tissue was withdrawn. Muscle samples were separated from any fatty tissue and divided into 25–50 mg portions. Approximately 20 mg of tissue was placed in ice-cold BIOPS buffer (60 mM K-MES, 35 mM KCl, 7.23 mM K_2_EGTA, 2.77 mM CaK_2_EGTA, 20 mM imidazole, 0.5 mM DTT, 20 mM taurine, 5.7 mM ATP, 15 mM PCr, and 6.56 mM MgCl_2_) for respiratory analysis. Portions designated for sectioning and microscopic analysis of the cross-sectional area were mounted on a cork with tragacanth gum and frozen in isopentane cooled in liquid nitrogen. Portions designated for mRNA or protein analyses were snap-frozen in liquid nitrogen. All frozen samples were stored at −80°C for analysis following completion of the study.

#### Limb subcutaneous fat measurements.

Subcutaneous fat was measured using a LOGIQ S8 diagnostic ultrasound machine (GE Healthcare, Chicago, IL) while the subject was lying in supine position. Images were collected using a ML6-15‐D matrix linear transducer probe (LOGIQ S8; GE Healthcare, Chicago, IL). Scanning depth (3 cm), frequency (8 MHz), focal position, and time‐gain compensation were kept constant. To ensure even probe pressure and avoid compressing the subcutaneous tissue, a generous layer of gel was applied to the skin, and the probe was held lightly on top of the gel without compressing the skin. Images were taken on both legs to investigate any possible effect of mechanical stimulation on subcutaneous fat thickness. Images of the muscle’s fascial border were captured and analyzed by a blinded investigator using software on the LOGIQ S8 machine.

#### RNA-Seq.

Total RNA was isolated using Trizol reagent (ThermoFisher) according to the manufacturer’s instructions. Three samples from the PM group (2 men and 1 woman) and three samples from the control group of *study 1* (2 men and 1 woman) were selected based on RNA quality (i.e., purity) and sent to Novogene for RNA-Seq analysis. In brief, mRNA was purified from the total RNA using poly-T oligo-attached magnetic beads. After fragmentation, the first strand cDNA was synthesized using random hexamer primers, followed by the second strand cDNA synthesis using either dUTP for directional library or dTTP for nondirectional library. The library was checked with Qubit and real-time PCR for quantification and bioanalyzer for size distribution detection. Quantified libraries were pooled and sequenced on Illumina platforms. The mapped reads of each sample were assembled by String Tie (v1.3.3b) in a reference-based approach. Feature Counts v1.5.0-p3 was used to count the read numbers mapped to each gene. Then FPKM of each gene was calculated based on the length of the gene and reads count mapped to this gene for visualization purposes. Differential expression analysis of the samples was performed using the DESeq2R package (1.20.0). DESeq2 provide statistical routines for determining differential expression in digital gene expression data using a model based on the negative binomial distribution. The resulting *P* values were adjusted using the Benjamini and Hochberg’s approach for controlling the false discovery rate. Genes with an adjusted *P* value < 0.05 found by DESeq2 were assigned as differentially expressed. Gene ontology (GO) enrichment analysis of differentially expressed genes was implemented by the cluster Profiler R package, in which gene length bias was corrected. GO terms with corrected *P* value less than 0.05 were considered significantly enriched by differential expressed genes.

#### Mitochondrial respiration.

Bundles of skeletal muscle fibers (2–5 mg) were blotted, weighed, and gently teased apart using fine-tipped forceps to partially separate fibers without removing them from the fiber bundle. Each fiber bundle was rotated for 30 min at 4°C in BIOPS buffer containing 50 μg/mL saponin to selectively permeabilize cell membranes. Fiber bundles were then rinsed for 15 min in ice-cold MiR05 buffer (110 mM sucrose, 60 mM potassium lactobionate, 2 mM MgCl2, 20 mM taurine, 10 mM KH2PO4, 0.5 mM EGTA, 20 mM HEPES, and 1 g/L bovine serum albumin). Following the rinse step, bundles were placed inside the Oroboros Oxygraph-2k (O2k) with MiR05 buffer, set at 37°C with the stir bars spinning at 750 revolutions/min. Supplemental oxygen was added to each chamber to maintain O_2_ concentrations between 500 and 200 μM throughout the experiment. Substrates were added using the following two titration protocols to measure mitochondrial respiration:

#### Titration protocol 1.

Glutamate (10 mM final concentration), malate (2 mM), and succinate (10 mM) were added to stimulate LEAK respiration (GMS), which is described by Pesta and Gnaiger (21). Following this, ADP was titrated in steps (12.5–10,000 µM) to determine respiratory kinetics and maximal coupled respiration (GMS_p_). Cytochrome c (10 µM) was then used to ensure that the outer mitochondrial membrane was intact. Any increase >10% following the addition of cytochrome c resulted in those data being excluded from analysis. Antimycin A (2.5 µM) was added to inhibit CIII and determine background oxygen consumption. Any remaining signal was subtracted from the previous measures. Due to the variability of weighing and teasing apart muscle fibers, measures were made in duplicate, where possible, to ensure accurate results.

#### Titration protocol 2.

Octanoylcarnitine (0.5 mM) and malate (2 mM) were used to stimulate LEAK respiration (OcM), followed by ADP (2.5 mM), to induce maximal fatty acid oxidation (OcM_p_). Succinate (10 mM) was then added (OcMS_p_), followed by cytochrome c (10 µM), to ensure mitochondrial membrane integrity and antimycin A (2.5 µM) to determine background oxygen consumption.

#### H_2_O_2_ emission.

H_2_O_2_ emission rates were determined using the O2k fluorimetry module. Amplex Red (10 µM) and horseradish peroxidase (1 U/mL) were added before introducing the fiber bundles to the chambers. H_2_O_2_ standard was then added to each chamber in 0.1 µM steps to calibrate the sensors. H_2_O_2_ emission from the mitochondria was then measured simultaneously with O_2_ consumption.

#### Western blotting.

Western blot samples were made up from muscle homogenate supernatant at equal protein concentrations in Laemmli buffer and heated at 45°C for 5 min. Proteins were either run on a Protein Simple WES platform (Integrin beta1, GADH) as described previously (22), or run on sodium dodecyl sulfate-polyacrylamide gels (4% stacking and 15% resolving) for 30 min at 100 V followed by 60 min at 150 V (catalase). Thirty micrograms of protein were loaded in each well of the gel. Separated proteins were transferred to PVDF membranes (1620112; Bio-Rad) at 100 V for 60 min in ice-cold transfer buffer. Following the protein transfer, the membranes were stained for total protein to ensure equal loading using Ponceau S BioReagent (P3504, Sigma-Aldrich). Each membrane was blocked for 60 min in 5% bovine serum albumin in Tris-buffered saline, 0.1% Tween 20 (TBST), followed by an overnight application of catalase antibody (1:500) or SOD2 antibody (1:1000) diluted in 1% bovine serum albumin in TBST at 4°C. Membranes were washed in TBST, incubated for 60 min at RT in anti-mouse IgG secondary antibody (1:10,000; IRDye; 926-65010), and diluted in 1% bovine serum albumin in TBST. Membranes were again washed in TBST and imaged using direct infrared imaging (Odyssey CLx, LICOR Biosciences). All protein values were normalized within subject to their own baseline measure and expressed as a fold difference from baseline, normalized to a housekeeping gene (GAPDH).

#### qRT-PCR.

Muscle tissue samples isolated from subjects pre- and 6 wk postintervention were transported via liquid nitrogen from −80°C to be weighed in a 500 μL TRIzol solution. Tissue samples ranged from 20 to 60 mg. Manual homogenization followed. About 200 μL chloroform were subsequently added prior to a series of centrifuge cycles and alcohol washes. RNA extracts were measured for concentrations varying from 250 to 1,000 μg/μL and standardized to 500 μg in creating cDNA with iScript cDNA synthesis kits. Spectroscopy measurements were additionally used to confirm RNA purity. The cDNA was mixed with various primers, SYBR Green Master Mix and nuclease-free water for a total of 20 μL increments in 96 well plates. Beta-2-microglobulin (*B2M*) acted as a standard to normalize all Ct values from various genes, as no changes were found in this gene from the pre- to 6 wk postbiopsy time points. Two other unrelated mRNAs, *GAPDH* and *RPLP0*, were measured and found to increase at the postbiopsy time points. Data collection occurred in mean Ct triplicate values within 1.5 standard deviations of each other. Primers were tested for adequate efficiency prior to usage. All matched paired subject samples were measured simultaneously on a plate to prevent error across experiments.

#### Histology.

Eight-micrometer cross sections of muscle samples were cut at −27°C and mounted to slides. For immunohistochemical stains, sections were fixed in 4% paraformaldehyde for 5 min. Sections were incubated in primary antibodies overnight at 4°C. Following incubation with primary antibodies, samples were rinsed three times in 1X phosphate-buffered saline (PBS) and incubated in secondary antibodies for 45 min at room temperature. Samples were then rinsed again, mounted in fluoroshield mounting medium (#F6182; Sigma-Aldrich), and imaged using Olympus IX73 microscope and Olumpus XM10 camera. Antibodies included rabbit anti-laminin (1:300; ab11575; Abcam), mouse anti-pax7 (1:100; Pax7; DSHB), mouse anti-myosin heavy chain I (1:20; BA-D5; 188 μg/mL; DSHB), and mouse anti-CD31 (1:100; Agilent, Santa Clara, CA). Secondary antibodies included anti-rabbit (1:100; #111-545-003; Jackson ImmunoResearch Laboratories, Inc.) and anti-mouse (1:100; #115-605-207; Jackson ImmunoResearch Laboratories, Inc.). Myofiber area was determined from 50 type I and 50 type II fibers at every time point for each subject. All images were analyzed using Olympus CellSens software (Olympus, Tokyo, Japan). For the Picosirius red stain, sections were first fixed, then incubated in a solution of sirius red dissolved in saturated picric acid. Following staining, tissue was rinsed in acidified water to remove unbound dye and dehydrated through graded ethanol before coverslipping. Sections were imaged under both light and polarized light conditions. Under polarized light microscopy, Picrosirius red enhances birefringence; collagen fibers appear bright red, orange, or yellow against a dark background, whereas noncollagenous structures remain minimally stained. This staining method has been validated as a method to determine collagen packing density.

#### Muscle functional measures.

Maximal knee extensor muscle strength was measured using a multijoint dynamometer (Biodex Medical Systems). To evaluate isometric strength, subjects were instructed to perform a 5-s maximal voluntary isometric contraction against a brace set at a 70° angle. For the isokinetic strength testing of the knee extensor muscles, participants performed three maximal voluntary contractions against a brace at five different velocities: 30°/s, 75°/s, 150°/s, 220°/s, and 300°/s, each separated by 90 s of rest. The range of motion for isokinetic knee extension measurements was set at 75° (25°–100°). Participants received verbal encouragement during exertion.

#### Microdialysis.

Microdialysis was performed to assess subcutaneous adipose tissue lipolysis and local perfusion. Following the PM intervention, subjects rested in a supine position, and microdialysis probes were inserted into the subcutaneous adipose tissue overlying the vastus lateralis of each thigh. Probes were continuously perfused at 1.0 μL·min^−1^ with lactate-Ringer solution containing ethanol (10% vol/vol) to enable estimation of relative changes in local blood flow via the ethanol outflow/inflow ratio. After a stabilization period, dialysate was collected in sequential 30-min fractions for 90 min. Samples were immediately frozen and stored at −80°C until analysis. Glycerol concentration was used as an index of lipolysis, and ethanol recovery was used to assess perfusion. Glycerol (GLY) relative recovery was calculated as the ethanol outflow/inflow ratio according to the following equation:

$$\text{In vivo}\;\text{GL}{\text{Y}}_{\text{relative recovery}}=\left(\text{In vitro}\;\text{GL}{\text{Y}}_{\text{relative recovery}}/\text{Invitro}\;\text{EtO}{\text{H}}_{\text{relative recovery}}\right)\times \;\left(1-\text{In}\;\text{vivo}[\text{EtOH}{]}_{\text{dialysate}}/[\text{EtOH}{]}_{\text{perfusate}}\right)$$

Adipose interstitial glycerol concentration [GLY] was calculated according to the following equation:

$$\text{In vivo}[\text{GLY}{]}_{\text{interstitial}}=\text{In}\;\text{vivo}[\text{GLY}{]}_{\text{dialysate}}/\text{In vivo}[\text{GLY}{]}_{\text{relative recovery}}$$

#### Statistics.

Normality of each dataset was determined using a Shapiro–Wilk test. When data were not normally distributed, they were log transformed. This was only the case for the MMP/TIMP data. We used a mixed model analysis of variance (ANOVA) to examine the main effects of time (pre vs. post) and group (SHAM vs. massage) in conjunction with the interaction of group × time for all variables. For qRT-PCR and Western blot data, analyses were done on the normalized Ct or density values, and data are displayed relative to the prebiopsy time point in graphs. If the fixed-effect test indicated a significant *P* value for a main or interaction effect, a Tukey honestly significant difference (HSD) test was performed post hoc. All data were analyzed using JMP software (JMP Pro, v. 16.0.0, SAS Institute Inc.), and significant differences were tested with an alpha level of 0.05.

## RESULTS

### Six-Week Repeated PM Study

To understand how repeated application of mechanical stress impacted skeletal muscle adaptation, we performed a 6-wk clinical trial, wherein subjects were treated with percussive massage (PM) for 30 min per day, three times per week. A schematic of the basic study design and subject anthropometric measures for this study can be found in Fig. 1*A*. Each group included both men and women, yet the study was not designed nor powered to detect sex differences for any outcome measure. Though neither group met the threshold for overweight (BMI > 25), there was a statistically significant difference in weight (*P* = 0.007) and BMI (*P* = 0.004) between the SHAM and PM groups, with the PM group being heavier and having a greater BMI compared with the SHAM group.

#### Tissue morphology.

To determine whether PM broadly induced changes in tissue morphology, we assessed morphological changes in the skeletal muscle, as well as the overlying subcutaneous fat layer. There were no differences over time or between groups for type I (group × time; *P* = 0.174), type II (group × time; *P* = 0.584), or total (group × time; *P* = 0.342) myofiber cross-sectional area (CSA; Fig. 1, *D* and *E*). In addition, there were no changes in fiber type distribution between groups or across time (data not shown). As the percussive force was transmitted through the subcutaneous fat layer, we also evaluated whether PM treatments altered the thickness of subcutaneous fat over the vastus lateralis muscle of each subject. Because the cohort of control subjects was part of a separate study that did not assess subcutaneous fat, we used the nonmassaged leg as an internal control for this measure. We found a significant effect of time (*P* = 0.008) and the limb × time interaction (*P* = 0.012) for subcutaneous fat thickness. Post hoc analysis showed that the treated thigh decreased significantly by 7.3% (*P* = 0.001) over time, whereas no such change occurred in the contralateral thigh (−0.8%; *P* = 0.991; Fig. 1*B*).

#### Whole muscle RNA-Seq.

**Figure 2.**
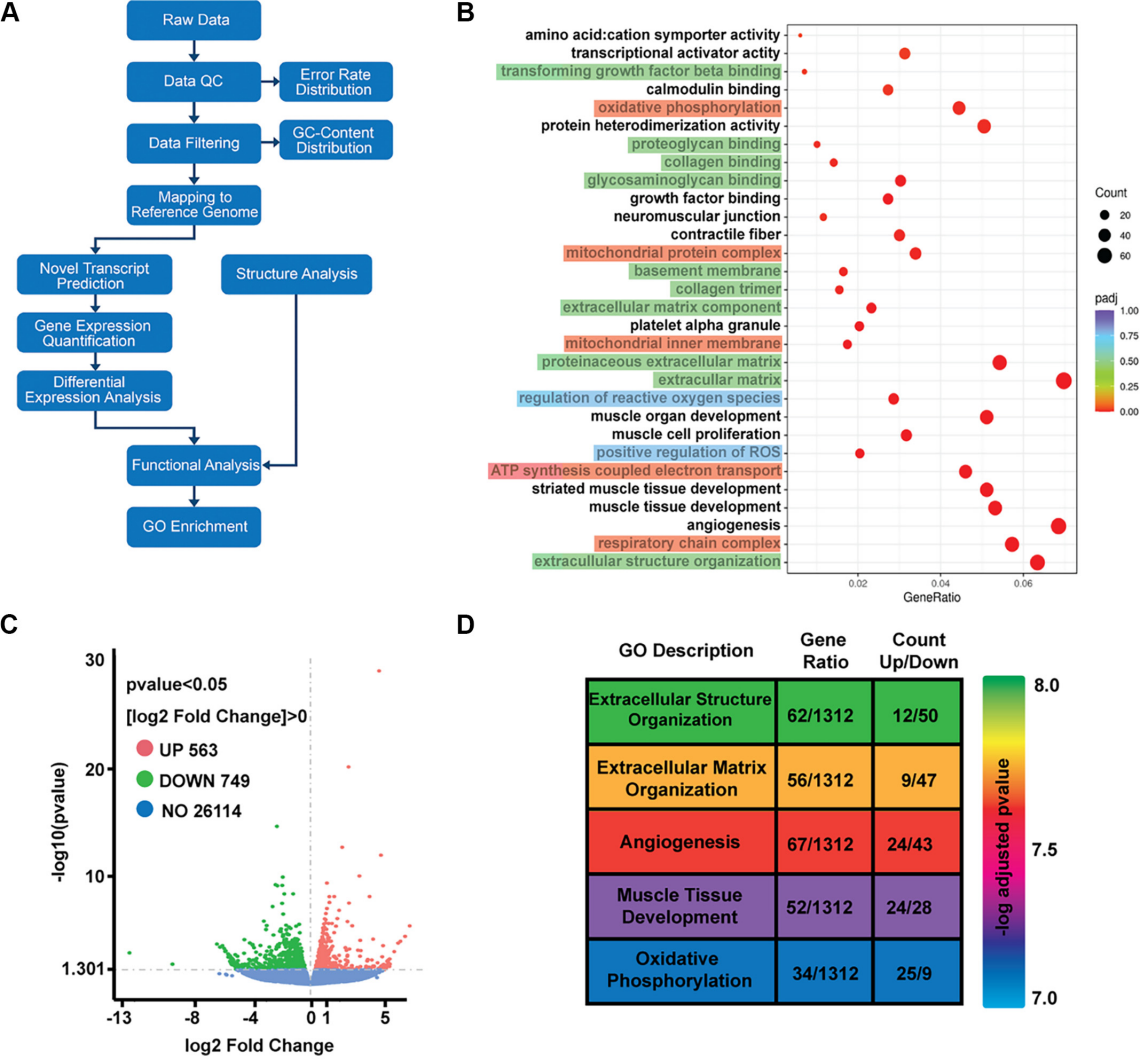
Whole muscle RNA-Seq following 6 wk of percussive massage (PM). *A*: schematic showing the RNA-Seq and bioinformatics analysis workflow. *B*: gene ontology enriched biological process analysis of differentially expressed genes in the PM group (*n* = 3) relative to the SHAM (*n* = 3) group. Highlighted terms indicate functionally related ontologies grouped by biological theme: extracellular matrix (green), mitochondrial processes (red), and reactive oxygen species-related pathways (blue). *C*: Volcano plot depicting the total number of differentially expressed transcripts in the PM group relative to the SHAM treatment group. *D*: gene ontology of the top ranked biological functions, with gene ratio and total number of genes altered in that ontology represented.

To develop testable hypotheses and identify transcriptomic networks that may be altered by PM, we next performed whole muscle RNA-Seq analysis on a small subset of three subjects in both the PM and SHAM groups. Overall, 563 and 749 genes were up- and downregulated, respectively, in the PM group relative to the SHAM group (Fig. 2*C*). To assess the biological pathways modulated by massage, we performed reactome pathway enrichment analysis on the differentially expressed genes. The top enriched pathways were predominantly related to extracellular matrix (ECM) remodeling, with additional contributions from vascular and metabolic elements (Fig. 2*B*). Among the most significantly enriched pathways were those involved in extracellular matrix structure and organization, angiogenesis, and oxidative phosphorylation (Fig. 2*D*). Highly enriched pathways within the extracellular matrix ontology included “Extracellular matrix organization,” “Integrin-mediated cell surface interactions,” and “ECM proteoglycans,” with transcripts associated with those groups generally being downregulated (Fig. 2*D*). Further gene ontology (GO) and pathway analysis confirmed that within the broader oxidative phosphorylation ontology, genes associated with complex I and complex IV were highly represented—the majority of those transcripts being upregulated (Fig. 2*D*).

#### Extracellular matrix remodeling.

**Figure 3.**
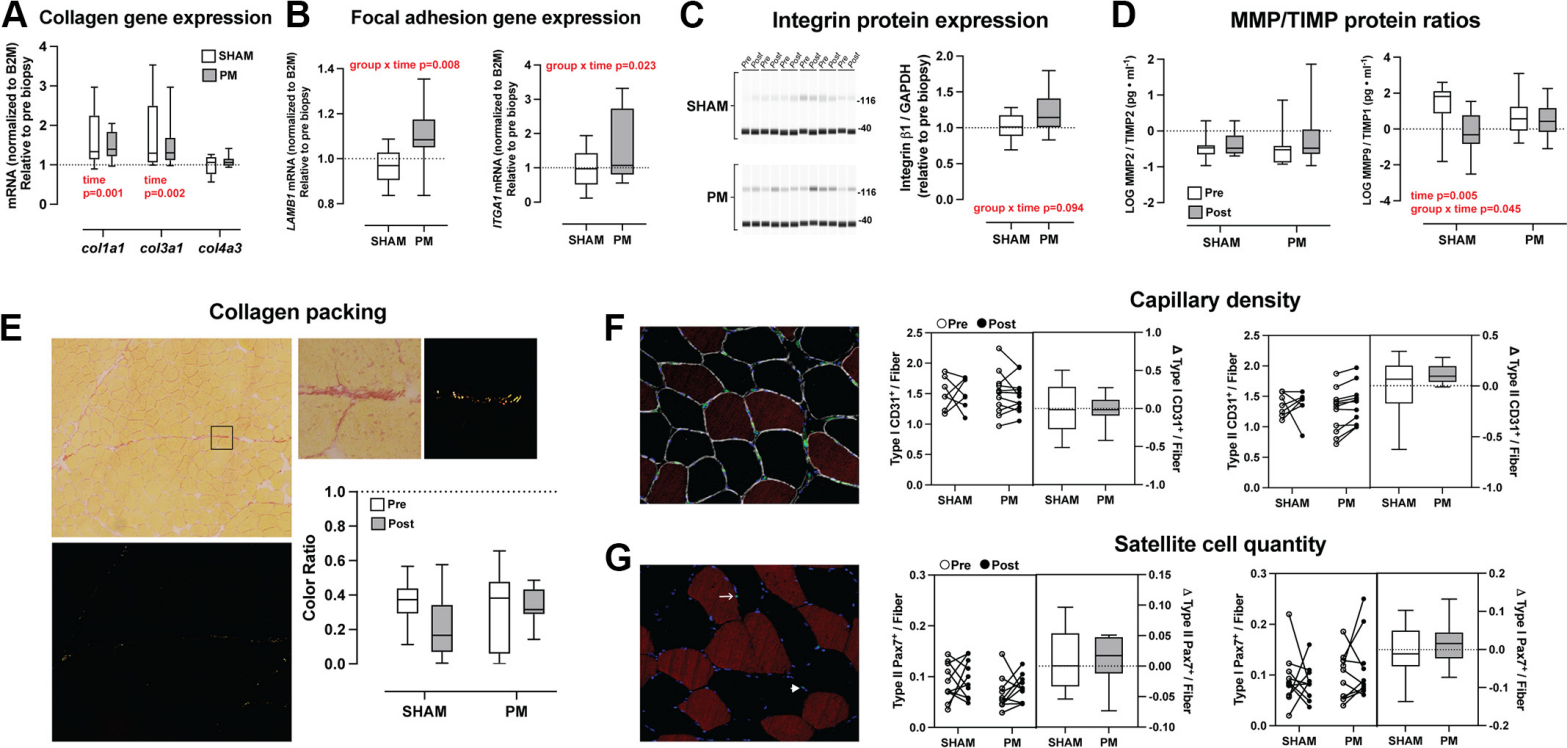
Extracellular matrix (ECM) adaptation following 6 wk of percussive massage (PM). *A*: expression of collagen I, III, and IV transcripts in the SHAM (*n* = 8) and PM (*n* = 11) groups, displayed as normalized ct values, relative to the preintervention time point. *B*: expression of laminin and integrin transcripts in the SHAM (*n* = 9) and PM (*n* = 11) groups, displayed as normalized ct values, relative to the preintervention time point. *C*: expression of integrin beta 1 subunit in the SHAM (*n* = 10) and PM (*n* = 10) groups, displayed as normalized band intensity values relative to the preintervention time point. The Western blot images depict a representative blot of 6 subjects in each group, showing both integrin (∼126kDa) and GAPDH (∼36kDa) bands. *D*: log-transformed concentrations of MMP2 and MMP9, shown as ratios relative to their respective inhibitors TIMP2 and TIMP1 in the SHAM (*n* = 9) and PM (*n* = 12) groups. *E*: analysis of cross sections stained for Picosirius red and analyzed under cross-polarized light to reflect collagen packing density, displayed as a pixel density ratio for green • red/orange/yellow^−1^. The representative image shows both the image obtained under normal light and cross-polarized light, with the black square showing a zoomed image of a perimysial region for more detail. *F*: representative image and quantification of capillary density associated with type I and type II fibers in the SHAM (*n* = 8) and PM groups (*n* = 11). white = laminin, red = MHC I, green = CD31, blue = DAPI. *G*: representative image and quantification of satellite cell abundance associated with type I and type II fibers, normalized to total fiber number in the SHAM (*n* = 10) and PM (*n* = 9) groups. Red = MHC I, green = CD31 or Pax7, blue = DAPI. Data for *A*–*D* are presented as box and whisker plots displaying distributions including median, interquartile range, and spread. Data for *F* and *G* are presented in a combination panel composed of a paired dot plot with individual subject pre- and postintervention values and a box and whisker plot displaying the distribution of change scores including median, interquartile range, and spread. *P* values are shown in red, where a significant (*P* < 0.05) main effect was found.

To follow up on the top two ontology networks identified in the transcriptome analysis, we next assessed whether 6 wk of PM resulted in evidence of extracellular matrix remodeling and angiogenesis. qPCR analysis of the major collagen isoforms found in muscle revealed a significant increase in *COL1A1* and *COL3A1* over time in both groups (*P* = 0.001 and *P* = 0.002, respectively) but no group or group × time interactions. Expression of the basement membrane-associated transcript *COL4A1* was not altered over time in either group (Fig. 3*A*). Despite the majority of ECM-related transcripts being downregulated in the RNA-Seq dataset (see Fig. 2), we noted that focal adhesion-related transcripts tended to be increased. To verify focal adhesion adaptation, we assessed laminin and integrin expression at both the transcript and protein level. Both *LAMB1* and ITGA1mRNA expression increased in the PM group relative to the SHAM group (group × time interaction of *P* = 0.008 and *P* = 0.023, respectively). Post hoc testing showed a 1.34-fold increase pre to post for *LAMB1* (*P* = 0.005) expression and a 1.5-fold increase in *ITGA1* (*P* = 0.049) expression, with no significant changes found pre-to post-PM in the SHAM group (Fig. 3*B*). ITGB1 protein content showed a trend for an increase over time relative to the SHAM group (group × time interaction of *P* = 0.094; Fig. 3*C*). As an indirect marker of ECM breakdown, we measured matrix metalloproteinase/tissue inhibitor of metalloproteainases (MMP/TIMP) ratios using a magnetic bead assay. There were no changes in the MMP2/TIMP2 ratio over time or between groups. We did find a significant time (*P* = 0.005) and group × time interaction (*P* = 0.045) for the MMP9/TIMP1 ratio. Post hoc testing showed that the MMP9/TIMP1 ratio decreased pre- to postintervention only in the control group (*P* = 0.004; Fig. 3*D*). Picrosirius red staining was used to visualize and assess collagen organization in the perimysial regions of biopsy cross sections (Fig. 3*E*). Under cross-polarized light, collagen appears birefringent, producing a spectrum of colors from green to red, whereas noncollagenous tissue remains dark. The hues are thought to reflect collagen packing density, with loosely packed fibers appearing green and densely packed fibers red/orange/yellow (23). Accordingly, we quantified the relative areas of green versus red/orange/yellow staining in perimysial regions of biopsy cross sections and found no differences in collagen packing between groups or over time. Finally, we found no differences over time or between groups for type I- or type II-associated capillary density or ECM-resident satellite cells (Fig. 3, *F* and *G*).

#### Mitochondrial respiration and H_2_O_2_ flux.

To understand whether repeated PM induces muscle mitochondrial adaptation, we next measured respiratory capacity of permeabilized muscle fibers from each group using in vitro respirometry. We used two separate titration/inhibitor protocols. Titration 1 was performed to get a measure of general mitochondrial respiratory capacity, whereas titration 2 was performed to determine mitochondrial respiratory function under fatty acid-supported conditions.

#### Titration 1.

**Figure 4.**
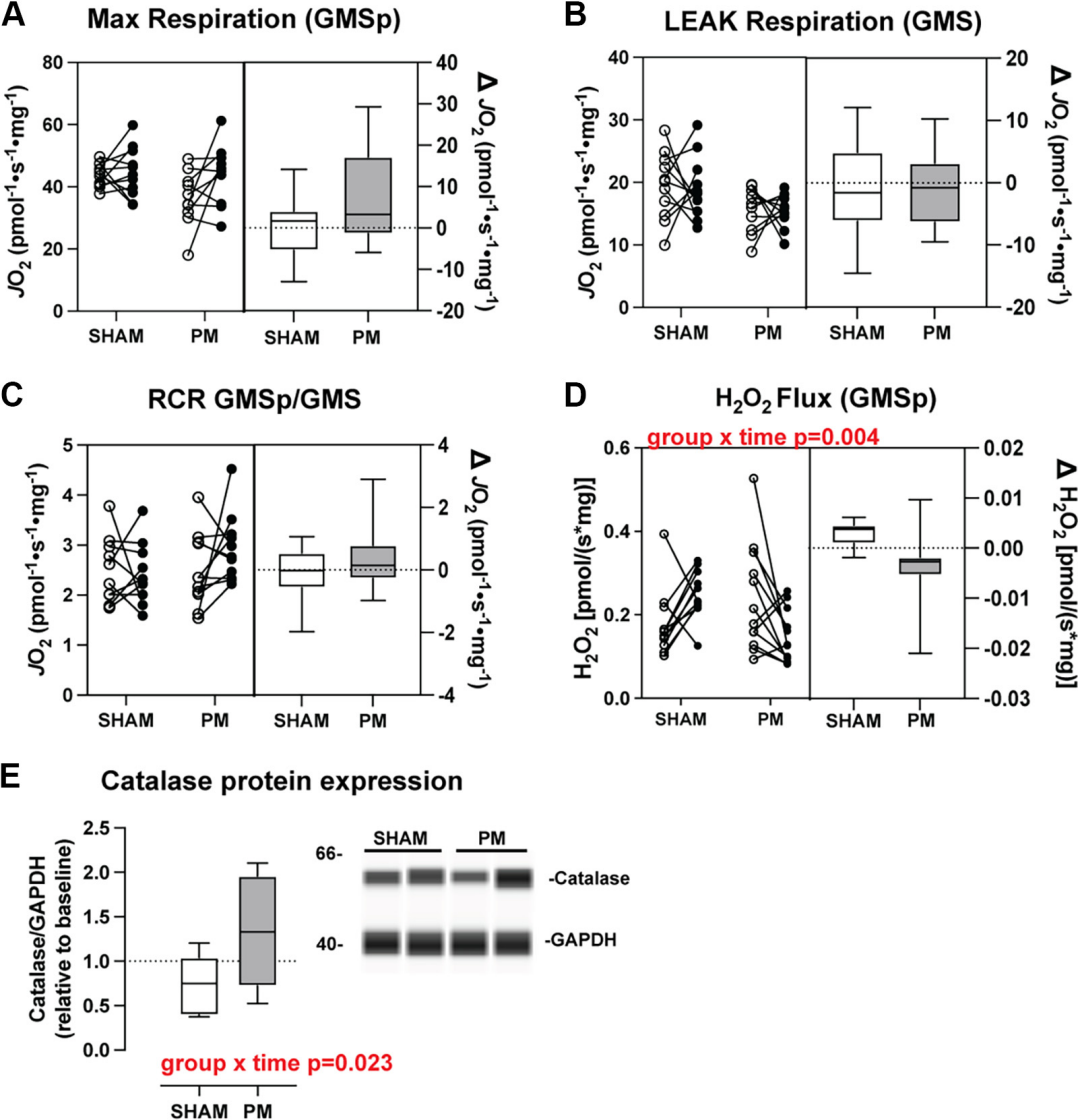
Changes in mitochondrial respiration following 6 wk of percussive massage (PM) or SHAM treatment. *A*: maximal mitochondrial respiration. *B*: maximal mitochondrial respiration in the absence of ADP (LEAK state). *C*: respiratory control ratio. *D*: maximal H_2_O_2_ flux in the presence of 12.5 µM ADP. *E*: change in the protein content of catalase, displayed as normalized relative band intensity values relative to the preintervention time point, and a representative capillary-based (WES) Western blot. *n* = 10 or 11 for the SHAM group and 11 or 12 for the PM group. Data are presented in combination panels composed of a paired dot plot with individual subject pre- and postintervention values and a box and whisker plot displaying the distribution of change scores including median, interquartile range, and spread. *P* values are shown in red, where a significant (*P* < 0.05) main effect was found.

For maximal coupled respiration supported by glutamate, malate, and succinate in the presence of ADP (GMS_p_), we found no significant differences over time or between groups (Fig. 4*A*). Likewise, we found no differences across time or between groups for LEAK respiration (glutamate, malate, and succinate in the absence of ADP; Fig. 3*B*). We also found no differences in intrinsic changes in coupling efficiency of the mitochondria, as indicated in the ratio of maximal coupled respiration (GMS_p_) to LEAK (GMS) respiration (RCR; Fig. 4*C*). We next assessed H_2_O_2_ flux under maximal coupled conditions and found a significant group × time interaction (*P* = 0.004). Post hoc testing indicated a significant 64% decrease (*P* = 0.016) in H_2_O_2_ emission in the PM group, with no change in the SHAM group (*P* = 0.071; Fig. 4*D*). As PM resulted in decreased H_2_O_2_ flux, we conducted further experiments to determine whether the effect may have been mediated by changes in catalase expression. Correspondingly, we found a significant group × time interaction (*P* = 0.023), whereby catalase protein expression increased in the PM group (*P* = 0.021) over time, with no significant change in the SHAM group (*P* = 0.101; Fig. 4*E*).

#### Titration 2.

**Figure 5.**
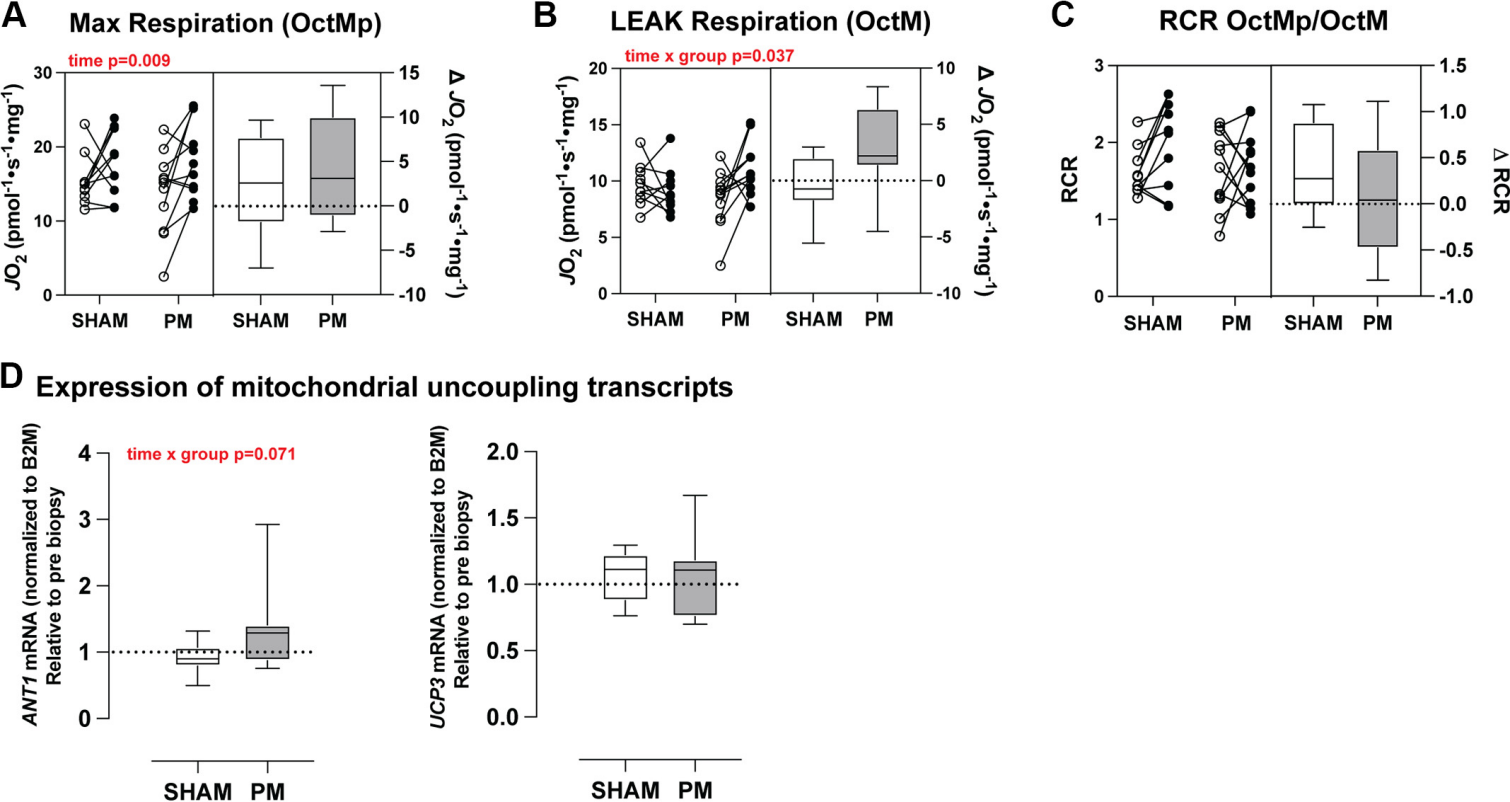
Changes in mitochondrial respiration under fatty-acid supported conditions following 6 wk of percussive massage (PM) or sham treatment. *A*: maximal fatty acid-supported respiration. *B*: fatty acid-supported respiration in the absence of ADP (LEAK state). *C*: respiratory control ratio under fatty acid-supported conditions. *D*: mRNA expression, as measured by qRT-PCR shown as normalized ct values, relative to the preintervention time point for transcripts associated with mitochondrial uncoupling (*ANT1* and* UCP3*). *n* = 9–11 for the SHAM group and 11 or 12 for the PM group. Data are presented in combination panels composed of a paired dot plot with individual subject pre- and postintervention values and a box and whisker plot displaying the distribution of change scores including median, interquartile range, and spread. *P* values are shown in red, where a significant (*P* < 0.05) main effect or trend was found.

When mitochondrial respiratory capacity was assessed under fatty acid-supported conditions (octanoylcarnitine, malate, ADP; OCtM_p_), we found a significant main effect of time (*P* = 0.009). Post hoc pairwise comparison testing confirmed a significant increase in fatty acid-supported respiration in the PM group only (*P* = 0.024; Fig. 5*A*). In the absence of ADP (LEAK), we found a significant interaction (*P* = 0.037), whereby PM increased leak respiration over time (*P* = 0.027), with no change in the SHAM group (Fig. 5*B*). There were no significant differences in RCR for fatty acid supported respiration, indicating that alterations in coupled fatty acid respiration are primarily due to changes in LEAK respiration (Fig. 5*C*). To follow up on potential mechanisms to explain alterations in fatty acid-supported LEAK state respiration, we next explored the hypothesis that PM alters LEAK state fatty acid-supported respiration via the upregulation of expression of transcripts associated with mitochondrial uncoupling proteins [adenine nuceleotide translocator 1 (ANT1) and uncoupling protein 3 (UCP3)]. For *ANT1* (*SLC25A4*) mRNA expression, there was a trend (*P* = 0.071) for the group × time interaction main effect, such that expression of *ANT1* increased over time compared with the SHAM group. There was no change over time or between groups for *UCP3* expression (Fig. 5*D*).

### Ten-Day Immobilization Study

**Figure 6.**
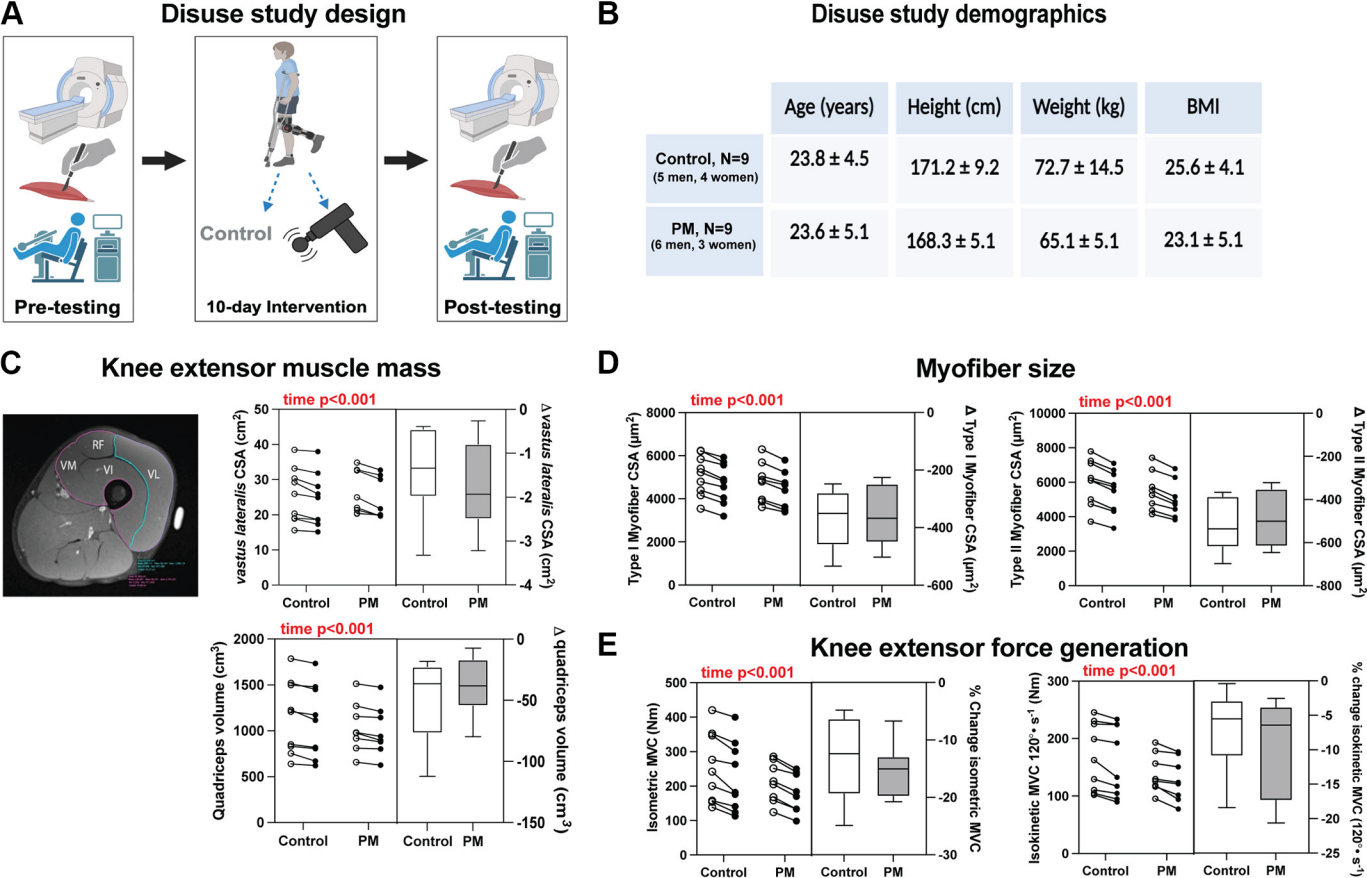
Immobilization study design, muscle size and function. *A*: schematic of the 10-day immobilization study design. *B*: demographics of the immobilization study for the control and percussive massage (PM) groups. *C*: a representative MRI image at 50% of femur length. Purple line shows total quadriceps cross-sectional area (CSA) tracing and blue line shows vastus lateralis CSA tracing. Data display changes in vastus lateralis cross-sectional area (CSA) and quadriceps volume. *D*: changes in type I and type II myofiber CSA in each group. *E*: changes in isometric and isokinetic maximal voluntary contractions (MVC) from pre- to postimmobilization. *n* = 9 control, *n* = 8 PM. Data are presented in combination panels composed of a paired dot plot with individual subject pre- and postintervention values and a box and whisker plot displaying the distribution of change scores including median, interquartile range, and spread. *P* values are shown in red, where a significant (*P* < 0.05) main effect was found.

We next asked the question of whether repeated PM might provide some structural or metabolic resilience in a pathophysiological context. Therefore, we recruited 10 men and 7 women to be part of a 10-day limb immobilization study. Subjects were randomly assigned to receive twice-daily PM or control treatments throughout the 10 days. A schematic of the study design and subject demographic data can be found in Fig. 6, *A* and *B*.

#### Immobilization-induced changes in muscle size and function.

No significant differences were observed in the baseline measures between groups. The cross-sectional area (CSA) of the vastus lateralis (VL) at 50% of the femur exhibited a similar decrease after 10 days of limb unloading in both groups (control group = −1.4 ± 0.9 cm^2^; PM group = −1.8 ± 1.0 cm^2^; *P* < 0.001; Fig. 6*C*). Quadriceps muscle volume also showed a similar decrease in both groups (control group = −49.8 ± 32.7 cm^3^; PM group = −38.4 ± 25.5 cm^3^; *P* < 0.0001; Fig. 6*C*). Likewise, myofiber CSA in biopsy samples showed a similar decrease for both type I and type II fibers (*P* < 0.001) in both groups (Fig. 6*D*). We determined changes in knee extensor muscle strength through isometric and isokinetic dynamometry assessments. Both the control group (−13.07 ± 6.98%) and the PM group (−15.24 ± 4.59%) experienced similar reductions in isometric maximal voluntary contraction (MVC) (*P* < 0.001; Fig. 6*E*). Furthermore, both groups exhibited reductions in isokinetic MVC (*P* < 0.001) with no group effect or interaction between group and time (Fig. 6*E*).

Because 6 wk of PM improved some aspects of mitochondrial respiration, we next sought to determine whether it could preserve metabolic function under the catabolic stress of limb immobilization.

#### Titration 1.

**Figure 7.**
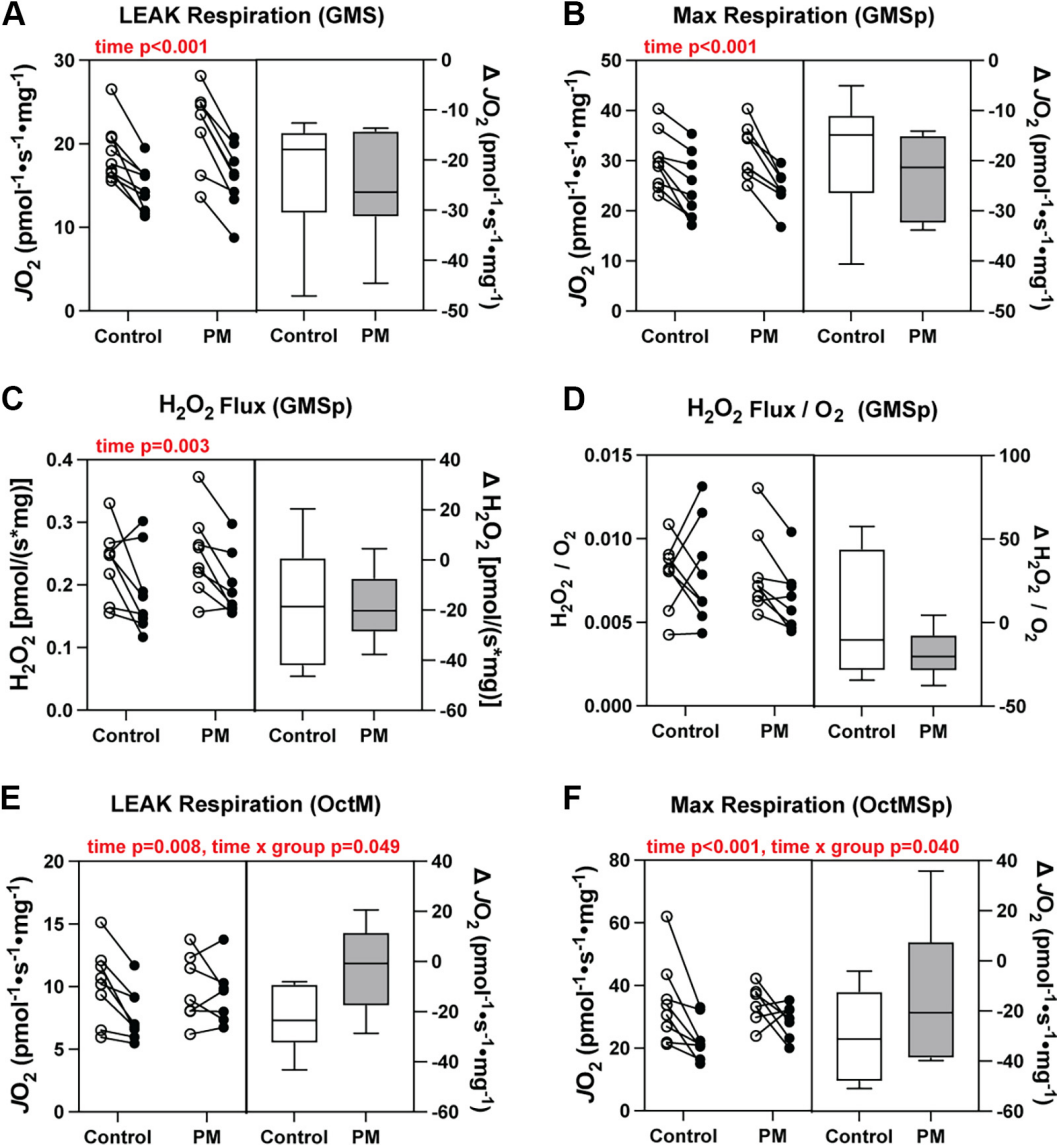
Changes in mitochondrial respiration following 10 days of limb immobilization in the control and percussive massage (PM) groups. *A*: LEAK respiration in the presence of glutamate, malate, and succinate. *B*: maximal mitochondrial respiration. *C*: maximal H_2_O_2_ flux in the presence of 12.5 µM ADP. *D*: maximal H_2_O_2_ flux normalized to oxygen consumption. *E*: LEAK respiration in the presence of octanoylcarnitine (Oct) and malate. *F*: fatty acid-supported respiration in the presence of 2.5 mM ADP and malate. *n* = 8 or 9 control, *n* = 8 PM. Data are presented in combination panels composed of a paired dot plot with individual subject pre- and postintervention values and a box and whisker plot displaying the distribution of change scores including median, interquartile range, and spread. *P* values are shown in red, where a significant (*P* < 0.05) main effect was found.

We found a significant decrease (*P* < 0.001) in LEAK respiration and maximal coupled respiration in response to 10 days of limb immobilization, with no differences between groups (Fig. 7, *A* and *B*). There were also no differences in coupling efficiency of the mitochondria, as indicated in the ratio of maximal coupled respiration (GMS_p_) to LEAK (GMS) respiration (RCR; data not shown). Maximal mitochondrial H_2_O_2_ flux also decreased in both groups (*P* = 0.003) with no differences between groups (Fig. 7*C*). When normalized to O_2_ consumption, there was no difference in H_2_O_2_ flux over time or between groups (Fig. 7*D*), indicating that decreases in H_2_O_2_ flux due to disuse is primarily driven by reductions in mitochondrial respiratory capacity.

#### Titration 2.

Measurement of LEAK respiration under fatty acid-supported conditions (octanoylcarnitine, malate; OctM) showed a significant effect of time (*P* < 0.008) and an interaction between the treatment group and time (*P* = 0.049; Fig. 7*E*). Post hoc analysis revealed that 10 days of limb unloading caused a significant decrease in LEAK respiration only in the control group (*P* = 0.009). Coupled fatty acid supported respiration, supported by octanoylcarnitine, malate, and ADP (OctMp), also showed a significant time effect (*P* < 0.001) and an interaction between group and time (*P* = 0.040; Fig. 7*F*). Post hoc analysis revealed only a significant reduction in coupled fatty acid respiration in the control group (*P* = 0.0008). There were no significant differences in RCR for fatty acid-supported respiration (data not shown).

### Acute Lipolysis Study

**Figure 8.**
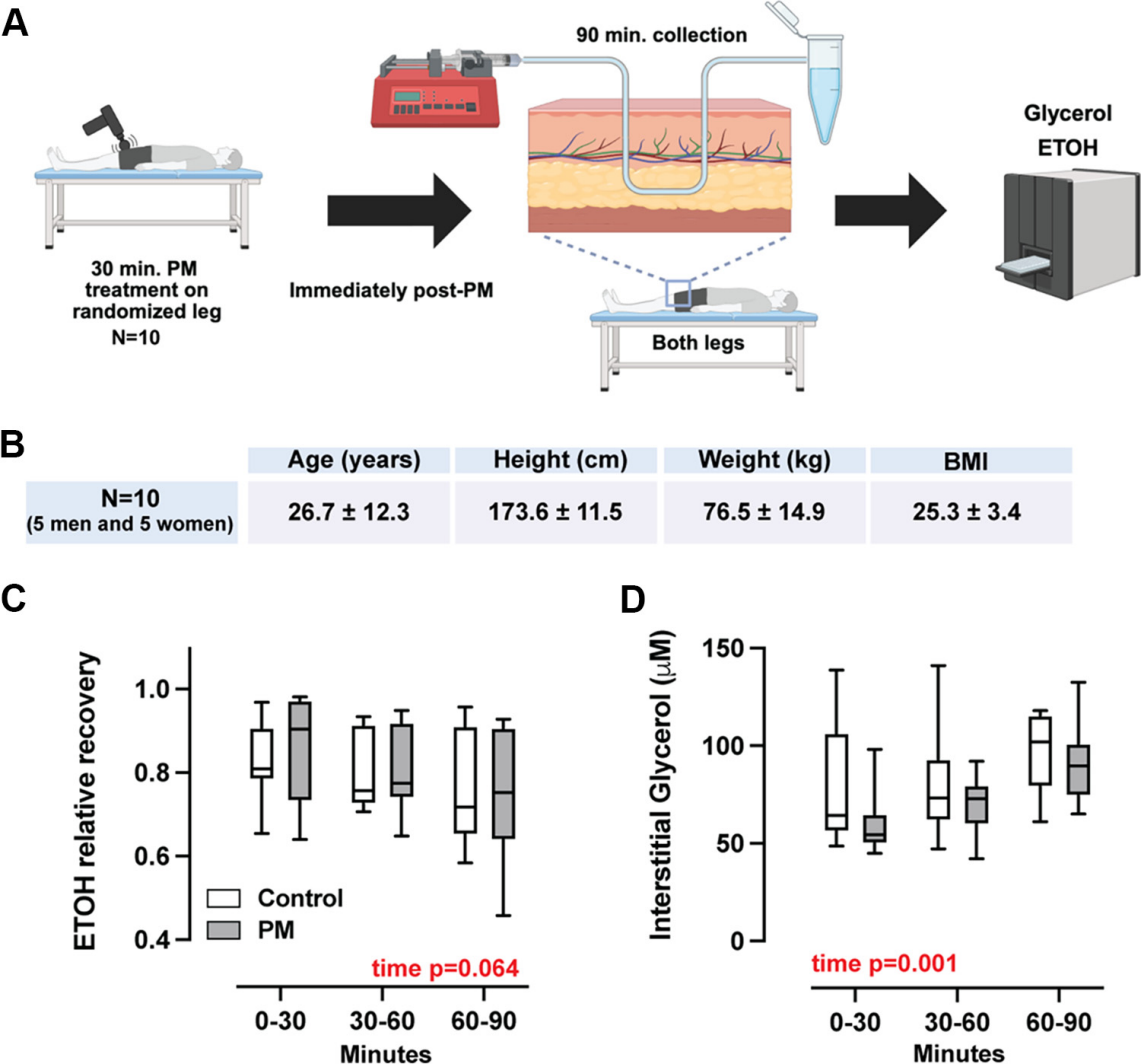
Lipolysis study design and interstitial adipose glycerol concentration*. A*: schematic of the study design to determine whether an acute 30-min percussive massage (PM) treatment induces subcutaneous adipose lipolysis. *B*: subject demographics of the lipolysis study. *C*: ethanol-relative recovery during the 90-min microdialysis collection period. *D*: interstitial subcutaneous adipose glycerol concentration during the 90-min microdialysis collection period. *n* = 10. Data are presented as a box and whisker plot displaying the distribution of change scores including median, interquartile range, and spread. *P* values are shown in red, where a significant (*P* < 0.05) main effect or trend was found.

Finally, because repeated PM reduced subcutaneous fat thickness (Fig. 1*C*) and both 6-wk and 10-day PM interventions improved elements of fatty acid-supported mitochondrial respiration (Fig. 5*B* and Fig. 7, *F* and *G*), we hypothesized that PM may stimulate local adipose tissue lipolysis, thereby driving a more fat-adapted phenotype in the underlying vastus lateralis muscle. To test this hypothesis, PM was applied to the knee extensor muscles of one randomized leg in 10 subjects (5 men and 5 women). Microdialysis samples were then collected from the subcutaneous adipose tissue overlying the vastus lateralis of both legs, and glycerol concentrations were measured as an index of lipolysis, with ethanol included to control for changes in local perfusion over the 90-min collection period (Fig. 8*A*). Subject demographics can be found in Fig. 8*B*. There were no significant changes in ethanol relative recovery over the 90-min collection period, though there was a trending main effect of time (*P* = 0.064), suggesting a subtle increase in subcutaneous perfusion, independent of the PM treatment, likely due to the microdialysis procedure. Likewise, interstitial glycerol concentration was increased over the collection period (main effect of time; *P* = 0.001), but was not different between the control leg and the leg that received PM. These data suggest that PM has no effect on subcutaneous adipose perfusion or lipolysis in the 90 min following a 30-min treatment.

## DISCUSSION

This study evaluated whether repeated mechanical stimulation via percussive massage (PM) could promote broad skeletal muscle adaptations under normal physiological loading conditions and whether such adaptations could potentially mitigate the metabolic and structural declines of short-term limb immobilization. Three main findings emerged. First, 6 wk of PM induced selective metabolic adaptations, characterized primarily by increased fatty acid-supported mitochondrial respiration in the LEAK state and reduced mitochondrial H_2_O_2_ emission. Second, PM induced modest transcriptional signatures related to extracellular matrix (ECM) remodeling, but did not broadly alter muscle morphology and did not protect against immobilization-induced losses in muscle size or strength. Third, acute PM did not increase adipose tissue lipolysis, despite repeated PM reducing subcutaneous fat thickness in vivo.

The most consistent adaptation observed across studies was enhanced mitochondrial lipid oxidative capacity. PM increased fatty acid-supported respiration under normal ambulatory conditions and partially preserved this function during 10 days of limb immobilization. This effect was most evident across both studies in the LEAK state, and importantly, it was reproducible across studies. Although it does not directly support ATP synthesis, LEAK-driven proton conductance is physiologically relevant due to its meaningful contribution to whole body oxygen consumption and substrate utilization (24, 25). Though there are no other PM studies in the literature to directly compare with the current finding, LEAK respiration has been studied in the context of other mechanical stimuli. For example, Kenny et al. (26) showed that a reduction in LEAK state respiration due to 10 days of bed rest was blunted by daily resistive vibration exercise. Moreover, it has been hypothesized for many years that mitochondria are likely highly mechanosensitive given their intricate linkage to cytoskeletal filaments and sensitivity to intracellular [Ca^2+^] (27). The two primary mechanisms known to induce proton leak in skeletal muscle are through uncoupling protein 3 (UCP 3) (28) and adenine nucleotide translocase (ANT1) (29). Of the two, ANT1 appears to play a more significant role in mitochondrial proton conductance within skeletal muscle mitochondria (29, 30). As our transcriptomic analysis indicated altered expression of the ANT1 transcript (*slc25a4*), we followed up on that finding using qPCR. We found a trend for increased *slc25a4* expression over time in the PM group relative the SHAM group, suggesting that increased expression of ANT1 may have contributed to the observed increase in LEAK state respiration.

Proton leakage plays a critical role in regulating the production of reactive oxygen species (ROS), and the activation of mitochondrial uncoupling proteins such as ANT1 has been associated with a reduction in ROS production (31). In accordance, 6 wk of PM treatment resulted in decreased H_2_O_2_ flux. This finding was accompanied by an increase in the protein content of the antioxidant enzyme catalase in the PM group. These two observations align, as catalase catalyzes the decomposition of hydrogen peroxide into water and oxygen, effectively lowering H_2_O_2_ emission. Although human studies in skeletal muscle are lacking, several key studies have shown improvement in a variety of serum oxidative stress markers, including an increase in catalase activity with a single session of both classical massage (32) and whole body mechanical vibration (33, 34). This is the first study that has explored the effect of any form of repeated mechanical stimulation in human skeletal muscle. Although the data seem to indicate that PM induces alterations that could be interpreted as beneficial, more research needs to be done to further explore the effect of PM on skeletal muscle oxidative stress markers.

As it pertains to the leg immobilization, although PM did not prevent atrophy or strength loss, the preservation of fatty acid-supported respiration may have some functional relevance. Impaired mitochondrial fatty acid oxidation (FAO) is a frequently observed feature of disuse and inactivity models (35), where decreases in FAO capacity accompany intramyocellular lipid accumulation and metabolic dysfunction (36). The specific mechanism for PM’s effect on fatty acid-supported respiration is unclear, as there are limited data directly linking mechanosensitive signaling pathways to muscle fatty acid oxidation. Nevertheless, there is some precedent in the literature for different forms of isolated mechanical stress (e.g., vibration) to impact lipid metabolism. For example, 12 wk of whole body vibration upregulated skeletal muscle lipid metabolic enzymes and decreased intramuscular lipid deposition in a metabolomic assessment of aged mice (37). Repeated mechanical vibration has also been shown to activate AMPK and increase CPT1 expression in rodent skeletal muscle (20).

Given the reproducible observation across studies that PM enhanced fatty acid-supported respiration, we hypothesized that this effect was perhaps related to the reduction in knee extensor subcutaneous fat in the 6-wk intervention trial. We suspected that repeated PM could have potentially generated a local lipolytic response that, when compounded over 6 wk, could have reduced fat mass and driven a fat-adapted phenotype in the underlying muscle. Therefore, we tested this hypothesis in a small acute study using a microdialysis approach. Contrary to our prediction, we did not detect a change in interstitial glycerol concentration relative to a control condition, suggesting that the fat-loss effect of repeated PM is unlikely to be mediated by lipolysis. Other potential mechanisms that may underlie the reduced fat thickness observation could include possible reductions in water content due to alterations in blood flow or lymphatic drainage or reduced ECM volume. Alternatively, PM may have influenced lipid handling within skeletal muscle independently of adipose tissue. For instance, a trend toward increased ANT1 expression in the PM group suggests that repeated PM exposure could prime mitochondrial uncoupling mechanisms to match a slightly increased reliance on fatty acids as fuel. This mechanistic possibility is consistent with prior observations that skeletal muscle is metabolically plastic in response to subtle shifts in substrate availability (38), and that mitochondrial FAO can increase even in the absence of measurable changes in acute lipolytic flux (35).

The transcriptomic and molecular data from the 6-wk intervention also indicated that repeated mechanical stimulation via PM modulates discrete elements of the extracellular matrix (ECM), producing a remodeling signature with no evidence of overt structural expansion. Pathway enrichment analysis in a very small sample of subjects indicated potential downregulation of broad ECM and proteoglycan ontologies, alongside upregulation of focal adhesion-related transcripts, suggesting a shift toward a more mechanically responsive matrix rather than a net increase in matrix deposition. Follow-up analysis in the full cohort of subjects indicated selective increases in integrin and laminin expression, in the absence of changes in total collagen abundance, collagen packing, capillarity, or satellite cell content. The data fall in line with a recent report in rats showing that 4 wk of manual massage increased skeletal muscle beta 1 integrin expression and improved colocalization of beta1 integrin and laminin (39). Such a response is consistent with the concept that focal adhesion complexes function as primary mechanotransducers, potentially improving myofiber sensitivity to repeated loading through cytoskeletal-ECM coupling. In addition, the attenuated reduction in MMP9/TIMP1 ratio suggests that PM may also modestly influence ECM turnover dynamics. Collectively, these findings support a model, in which repeated PM enhances ECM signaling capacity via the integrin-laminin axis.

Finally, prior animal and cell culture studies have suggested that mechanically induced cellular signaling pathways can stimulate protein synthesis, activate satellite cells, and potentially alter myofiber cross-sectional area (40). Recent rodent studies have also found that massage in the form of cyclic compressive loading can enhance the anabolic response to muscle reloading following a period of atrophy (41). However, that same model did not induce hypertrophy in unperturbed conditions (42). In this study, we likewise found that 6 wk of PM did not alter myofiber cross-sectional area, suggesting that PM alone is not a sufficient hypertrophic stimulus. In addition, PM was unable to prevent the decline in muscle mass, myofiber CSA, and force generation induced by 10 days of limb immobilization. This finding is consistent with a recent rodent study that failed to prevent muscle atrophy using cyclic compression loading during 7 days of hindlimb suspension (41). Collectively, the data suggest that isolated mechanical loading in the form of PM is not an effective strategy for preventing the decline in muscle mass and force producing capacity during a period of muscle disuse.

In summary, repeated PM elicited selective adaptations in skeletal muscle, characterized by enhanced mitochondrial fatty acid oxidative capacity, reduced mitochondrial oxidant emission, and modest remodeling within ECM elements. However, these metabolic and ECM-related adaptations were not sufficient to prevent the structural and functional decline in muscle size and strength during unloading. Moreover, PM treatment resulted in a marked reduction in subcutaneous fat thickness of the treated thigh. Although we were unable to identify a specific mechanism for this effect, our acute PM experiment raises doubt that the fat loss occurred via a lipolytic mechanism. The collective findings suggest that isolated mechanical stimulation can act as a targeted modulator of mitochondrial and ECM biology, but does not recapitulate the broader, multisystem adaptations characteristic of dynamic muscle contractions. Thus, PM may be best positioned as a complementary metabolic adjunct—rather than a stand-alone countermeasure—to support muscle health in ambulatory conditions or during periods when traditional loading is not feasible.

## Data Availability

All data supporting the results are reported in the manuscript. As such, there are no supporting data that have been archived or placed in a public repository.
